# Dr. Bennet Dowler

**Published:** 1853-09

**Authors:** 


*Dr. Bennet Dowler.*—The following, credited to the New Orleans Delta, we find in the Virginia Med and Surg. Journal:

"We are happy to learn that a petition, recommending Dr. BENNET DOWLER for a Foreign Consulship, was signed by all the members of the City Council, in their session night before last. Few persons will go before the President for an office with such high recommendations as have been given to this distinguished savant, whose scientific researches have reflected so much credit upon our city, and so much honor upon himself.

